# The Preparation of Pure Chloroform

**Published:** 1866-01

**Authors:** 


THE PREPARATION OF PURE CHLOROFORM.—M. Adrian (Bull. de Thér, lxvii, p. 357) referring to the fact that impure chloroform has but little anæsthetic power, and pure chloroform is but seldom to be had, remarks, that among the many impurities, alcohol, hydrochloric acid, ether, different combinations of methyl, aldehyde, etc., some are easily detected, others, more similar to the chloroform, with greater difficulty. Chloroform is, according to Subeiren, free from alcohol, when it sinks in a mixture of equal parts of water and sulphuric acid; but only when considerably adulterated does it float on top. Others recommend chromic acid as a reagent, which, when alcohol is present, gives rise to a green color; Letheby recommends albumen, which, in the presence of a very small quantity of alcohol, coagulates. But the most noxious adulterations are the very ones most difficult to detect by the ordinary reagent, and to separate. Thus Adrian found, although he had apparently pure chloroform, free from the admixtures mentioned, that its boiling point and its density did not correspond to those of the genuine chloroform of Liebig and Subeiren, in consequence, certainly, of the presence of other (chloric) combinations of methyl. Adrian, therefore, proposes the following process for the preparation of pure anæsthetic chloroform: 1. Washing it with water, to separate the alcohol, until chromic acid no longer reacts and recently prepared nitro-sulphuret of iron is no longer dissolved. By this means the aldehyde present is also removed. 2. Adding to the chloroform so treated a weak solution of soda, to free it from chlorine, hydrochloric acid and the acids of chlorine of lower degree. 3. Digesting it for 24 hours with chloride of calcium, to remove the water. After this process it must evaporate without a residue, and can be used as an anæsthetic; but it is prudent first to determine its density and boiling point. If the latter still exceeds 61 degrees C. the chloroform must either be rejected as unfit for anæsthetic purposes, or be rectified once more.—*Schmidt's Jahrb*, 1865. *No.* 2.

